# Chemo-mapping and biochemical-modulatory and antioxidant/prooxidant effect of *Galium verum* extract during acute restraint and dark stress in female rats

**DOI:** 10.1371/journal.pone.0200022

**Published:** 2018-07-03

**Authors:** Anca D. Farcas, Augustin C. Mot, Cezara Zagrean-Tuza, Vlad Toma, Claudia Cimpoiu, Anamaria Hosu, Marcel Parvu, Ioana Roman, Radu Silaghi-Dumitrescu

**Affiliations:** 1 Faculty of Biology and Geology, Babes-Bolyai University, Cluj-Napoca, Romania; 2 Institute of Biological Research, Cluj-Napoca, branch of NIRDSB, București, Romania; 3 National Institute for Research and Development of Isotopic and Molecular Technologies, Cluj-Napoca, Romania; 4 Faculty of Chemistry and Chemical Engineering, Babes-Bolyai University, Cluj-Napoca, Romania; University of PECS Medical School, HUNGARY

## Abstract

*Galium verum* is a well-known medicinal plant which is used in various pathologies. *G*. *verum* extracts are characterized here using chromatography, where among the rich pool of phenolic acids of flavonoids two known anti-stress modulators, chlorogenic acid and rutin are identified in high quantities. Additionally, the extracts are characterized using a series of *in vitro* assays (EPR, DPPH, TPC and TEAC). Considering the chemical findings, the potential beneficial effects of the *G*. *verum* extract are explored here in a living organism exposed to stress induced oxidative damages. Thus, the biochemical-modulatory and antioxidant roles of two doses of *G*. *verum* extract are examined in animals exposed to acute restraint and dark stress (S). The animals were divided in groups [control, S, SG1 (exposed to 25 mg *G*. *verum* extract), SG2 (50 mg extract)]. Increased levels of lipid peroxidation (TBARS from 4.43 to 8.06 nmol/mL), corticosterone from 0.43 to 1.96 μg/dL and epinephrine from 44.43 to 126.7 μg/mL, as well as decreased antioxidant enzymes activities (SOD/CAT) were observed in the S group. The *G*. *verum* extract afforded a near-normal equilibrium within the biochemical parameters of animals exposed to RS, by reducing oxidative damage (TBARS at a 3.73 nmol/mL; CS at 0.90 μg/dL; EP at 63.72 μg/mL) and by restoring the antioxidant balance.

## Introduction

*Galium verum* L. (known by its popular name Lady’s bedstraw or yellow bedstraw) is a perennial plant, used in folk medicine as diuretics, choleretics, against diarrhea, spasmolithic and as a wound healing remedy. *G*. *verum* has also been shown to display beneficial effects against liver disorders and cardiovascular diseases [1]. *G*. *verum* extracts have been investigated for their chemical composition [2] and for the antioxidant activity, in the case of the methanol extract [1]. These activities were monitored using relatively standard procedures, with arguably limited direct biological relevance.

Daily exposure of various types of stress leads to stress-associated disorders, such as major depression, anxiety and gastrointestinal functional diseases. Stress-associated disorders are widely known to disturb cell homeostasis associated with biochemical changes and oxidative damage [3]. A stress model useful for investigating these types of changes is the restraint stress model, which stimulates numerous cellular pathways that lead to increased reactive oxidative species (ROS) production [4], thus enhancing the levels of oxidative stress as an otherwise normal phenomenon in the body [5]. The hypothalamic-pituitary-adrenal (HPA) axis is an essential component in response reactions to stress. Acute stress effects on HPA activity may be reflected in the biochemical picture of the organism, mostly in changes of plasma hormone stress levels, corticosterone (CS) and epinephrine (EP) [6]. Restraint stress alterations reflected by those parameters are also associated with altered antioxidant enzyme systems and with enhanced oxidative damage. Thus, these alterations are the negative effect of an increase in ROS production that may lead to membrane lipid injuries via peroxidation, as well as to protein oxidation and nucleic acid damage [7]. Against such forms of oxidative stress, the antioxidant scavenging enzymes (e.g. catalase CAT, superoxide dismutase SOD, glutathione peroxidase GPX) act in synergy other small-molecule antioxidants (e.g. vitamin C, E, glutathione)[8]. Antioxidant defense system protects the cell during mild stress exposure, but if the stress conditions become harder or if the organism suffers a nutritional deficiency, ROS concentrations increase significantly, to such extent that the antioxidant enzymes may be overwhelmed[8]. The possibility of employing supplementary antioxidant molecules and their ability to neutralize the ROS excess is a current topic of research [9,10]. In this respect, the plant kingdom is rich in natural antioxidants, mainly polyphenols, generally recognized to possess the ability of defending the organism from the damaging effects of free radicals [11–13].

The aim of the present study is to identify the presence of the main polyphenolic compounds and to evaluate antioxidant effects of *G*. *verum* extract on rats subjected to restraint stress. To the best of our knowledge, no previous experiments have been developed so far in order to investigate the effect of *G*. *verum* extracts on restraint-stressed rats, including biochemical profile (aspartate aminotransferase AST, alanine aminotransferase ALT, cholesterol, Total Proteins, Creatinine), oxidative stress (thiobarbituric acid reactive substances TBARS, CAT, SOD) and stress hormonal determinations (corticosterone and epinephrine).

## Material and methods

### Chemicals

The reagents included in standard assay packages with colorimetric and kinetic methods were obtained from BioMaxima S.A., Lublin, Poland. Thiobarbituric acid, FC (Folin-Ciocalteu) reagent, DPPH, sodium persulfate, toluene, acetone, ethanol, methanol, formic acid, PEG, dichlormethane, diphenylborinic acid aminoethylester (NP) and ethyl acetate were obtained from several companies (Sigma, Fluka, Merck). Standard samples of rutin, kaempferol, and chlorogenic acid were purchased from Sigma, Germany. Ferulic acid, galic acid and quercetin were obtained from Roth, Germany. Neutral formalin solutions were purchased from Chemical Company S.A., Iasi, Romania. All other chemicals and solvents used in the study were of analytical grade.

### Plant material

The aerial parts of *G*. *verum* L., which belongs to Rubiaceae family, in full blossom were collected from the vicinity of the Cluj-Napoca city (lat. 46°53’, long. 23°77’), located in west-central region of Romania. The species were identified based on an herbarium specimen (CL 666632) at the Botanical Garden “Alexandru Borza”, Babeș - Bolyai University, Cluj-Napoca, Romania by the taxonomist Dr. Mihai Pușcaș. Air-dried plants were ground into powder for analysis.

### Extraction procedures

The plant powder (150 g) was macerated into 450 mL hydro alcoholic solution (70% ethanol) for 7 days, at room temperature. After decanting the solution, the extract was collected, filtered and evaporated under vacuum. For the analytical evaluation of polyphenolic compounds, a small quantity of the extract was used for chromatographic analysis.

### Chromatographic analysis (HPTLC and HPLC)

Preliminary chromatographic separation was performed on HPTLC plates (10x10 cm with silica gel 60 F_254_, Merck, Darmstadt, Germany), which were eluted using a mobile phase consisting of toluene: acetone: formic acid (9:9:2, v/v/v). The elution was performed at room temperature, on a distance of 8 cm and using normal chromatographic twin chambers (Camag), which were pre-saturated with the mobile phase for 30 minutes. Samples of 5 μL of standard solutions (1mg/mL each) and 10 μL from the extracts were applied on the plate as 8 mm bands using the Linomat 5- Camag applicator, at 1,5 cm distance from the low edge of plate, with 80 nL/s speed. The developed plates were heated at 100°C for about 3 minutes and then were immersed in Natural Products solution (NP - 1g diphenylborinic acid aminoethylesterdissolved in 200 mL ethyl acetate) and then dried in cold air followed by another immersion in polyethylene glycol (PEG– 10 g polyethylene glycol 400 dissolved in 200 mL dichloromethane). Compound detection was obtained after immersion in the two solutions mentioned above, using visible and UV light at 254 and 366 nm. The images were obtained using the TLC visualizer—Digistore 2, Camag.

A HPLC-DAD method was optimized in order to separate and quantitatively determinate the compounds of interest, using an Agilent 1200 HPLC system (Waldbronn, Germany) equipped with an on-line vacuum degasser, quaternary pump, temperature controlled sample tray, automatic injector, a column thermostat compartment and a DAD detector. The chromatographic separations were run on a Zorbax C18 column (240 mm x 4.6 mm, 5 μm particle size) from Agilent Technologies. The injection volume was 5 μL (0.2 μm filtered extract), the column temperature was set to 30°C and the flow rate was 1.2 mL/min. Several preliminary tests were run by varying the experimental conditions (gradient, injection volume, wavelength detection, etc.). The optimum method consisted of a gradient elution using solvent A, 5 mM ammonium acetate pH 5.2 and solvent B as acetonitrile. The gradient was as follows: one minute at 5% B, 1–13 min from 5 to 30% B, 13–22 min isocratic at 30% B, 22–25 min from 30% to 90% B, 25–30 min from 90% to 100% B, 30–33 min isocratic at 100% B and 33–33.1 min back to 5% B where was kept until 35 min. Standards of chlorogenic acid, p-coumaric acid, cafeic acid, ferulic acid, rutin, hyperoside, isoquercitrin, quercetin, kaempferol, luteolin, and jatrorrhizine (an alkaloid) were employed, all of analytical grade purity for different commercial available sources. A calibration curve was constructed for five of the standards (those that were present in the extract and discussed in the Results section) at 22, 44, 88, 175, 350 μg/mL using the area of the peak by integration employed by the Agilent software. The limit of detection (LOD) and limit of quantification (LOQ) were determined by formula LOD = (3.3 × standard deviation of intercept)/calibration curve slope and LOQ = (10 × standard deviation of intercept)/calibration curve slope, respectively. The UV-Vis detection of the compounds was carried out using the DAD detector that measured the entire spectrum in 240–750 nm region every 1 s; the chromatograms were monitored at 242, 260, 280, 320, 340, 450 and 670 nm. Identification of the compounds was achieved by the chromatographic retention time (with a 0.3 s as tolerance) and by spectral similarities (higher than 99.9% was considered as positive). The chromatograms were exported and the graphs were developed in Excel. Acid hydrolysis of the extract was carried on in 2 M hydrochloric acid at 80°C for one hour, followed by filtration of a slight precipitate that was formed. The extract turned into deep green after hydrolysis, specific to decomposition and oxidation of chlorogenic acid and its derivates.

### Antioxidant and pro-oxidant activity evaluation

The antioxidant activity of the hydro alcoholic extract of *G*. *verum herba* was evaluated by three different methods: determination of total polyphenols content (TPC) using the Folin-Ciocalteu method, DPPH bleaching assay, and trolox equivalent antioxidant capacity (TEAC) assays as previously described [14]. The prooxidant reactivity of the extract was evaluated using a previously developed method that is described in detail elsewhere [15]. Briefly, the extract is treated with a catalytic amount of laccase that generates radicals from the components of the extract which are responsible of oxidation of the ferrous oxy hemoglobin (oxyHb) into the oxidized form (metHb). The kinetic profile and the rate of the oxyHb oxidation is a marker for the reactivity of the generated radicals.

### EPR spectroscopy measurements

The protocol for the Electron Paramagnetic Resonance (EPR)-based investigation is described fully described elsewhere [16], with slight modifications. Briefly, in a 1.5 mL Eppendorf tube containing 200 μL 35% aqueous solution of ethanol, 50 μL of extract were added, followed by 5 μL 0.5 M NaOH and rapid mixing and transfer into a glass capillary. The spectra were recoded using a Bruker EMX Spectrometer with continuous wave at X band (~9 GHz) at room temperature with following parameters: microwave power 9 mW, modulation amplitude 1 G, center field 3515 G, and sweep field of 50 G.

### Experimental animals

The experiments and animals’ welfare were conducted according to the Law no. 43/2014 on the protection of animals used for scientific purposes and to the 2010/63/UE Directive with the approval of the Ethics Committee from the Institute of Biological Research, Cluj-Napoca, Romania (Decision 1/28.02.2013). Healthy adult female white Wistar rats weighing 180±30 g, F1 generation, were purchased from the zoo base of the “Iuliu Hatieganu” University of Medicine and Pharmacy, Cluj-Napoca, and were housed in hygienic conditions, under a 12/12 h light/dark cycle, at 20°C, with no noise. Handling was performed quietly and gently. The standard diet and tap water were *ad libitum*.

### Study design

The animals were randomly assigned in 4 groups of 6 animals each, as follows: C (Control—unstressed animals), S (animals exposed to restraint stress and darkness, in the same time), SG1 (animals exposed to stress and treated with *G*. *verum* extract «25 mg/ kg b.w»), and SG 2 (animals exposed to stress and treated with *G*. *verum* extract «50 mg/ kg b.w»). Rats were exposed to restraint stress 3 h/day during 7 days. Besides, during stress conditions the animals were placed in a different, but dark room, which becomes a complementary stressor, known as light/dark stress [17], which leads to slightly increse levels of corticosterone[18]. The hydroalcoholic extract was administrated *via* oral gavage, every day at the same periods of time (10 a.m.- 13 p.m.), just before exposing the animals to stress conditions. We aimed to investigate if the *G*. *verum* extract could possibly counteract the harmful effects of reactive oxygen species generated by stress.

### Blood and tissue sampling

At the end of the experiment (7 days) the animals were sacrificed by decapitation under anesthesia with ketamin- xylazine cocktail (60 mg ketamine and 7.5 mg xylazine respectively/ kg b.w). Blood was collected and centrifuged in order to obtain blood serum, which was frozen at—70° C, until analysis. For histological analyses, the kidneys were taken after the animal dissection and fixed in neutral formalin solution (10%), for 24 h. Afterwards, they were embedded in paraffin and sectioned at 5 μm with a microtome (Reichert, Austria). The sections were histo-processed and colored using hematoxylin-eosine staining protocol.

### Biochemical parameters

Blood serum was used for the determination of total proteins (TP), creatinine concentrations (Crea), catalase activity (CAT), the concentration of thiobarbituric acid reactive substances (TBARS), aspartate aminotransferase activity (AST), alanine aminotransferase activity (ALT), corticosterone and epinephrine levels, using a semi-automatic biochemistry analyser (Evolution 2000) that executes photometric measurements and elaborates them according to the corresponding programs, and a UV-1600 PC spectrophotometer. TP, Crea, ALT and AST were measured using commercial standard assay packages with colorimetric (TP, Crea) and kinetic (ALT, AST) methods obtained from Biomaxima S.A (Lublin, Poland). CAT activity was determined by a kinetic method, using H_2_O_2_ as substrate, according to [19]. The decreasing absorbance of H_2_O_2_ was measured at 240 nm. SOD activity was measured based on a method described by [20]. TBARS levels were estimated according to a modified method of [21]. The reaction mixture was determined spectrophotometrically at 540 nm. Serum hormone levels were determined using ELISA commercial kits (Biomaxima, Poland), which included controls and standards for determination of the analyte.

### Statistical methods

All data are reported as the mean ± SEM. The Gaussian distribution was checked by the Shapiro-Wilk normality test. T test and one-way analysis of variance ANOVA, followed by Bonferroni’s Multiple Comparison test procedures were performed. Statistical significance was at p<0.05 (95% confidence interval). Statistical values were obtained using GraphPad Prism version 5.0 for Windows, GraphPad Software, San Diego California USA. Multivariate analysis of the spectral data was carried out using Principal Component Analysis (PCA) form the Statistica software (Statsoft, USA).

## Results and discussions

### Phytochemical analysis

The HPTLC data provide complementary information useful for the identification of polyphenols present in the *G*. *verum* extract. Firstly, compounds were identified based on the retention factor (R_F_) and on the fluorescence color. The extract was found to contain mainly chlorogenic acid, rutin and possibly quercetin, observable under visible light (S1 Fig). The extract does not contain gallic acid; the presence of ferulic acid (blue fluorescence) and kaempferol (blue-green fluorescence) is indicated by exposure at 366 nm light. Other compounds can be observed by means of red fluorescence (high R_F_ values) which could be assigned to chlorophylls (green in visible light) and stilbene compounds. Overall, based on HPTL analysis, chlorogenic acid and rutin appear to be the major compounds in the extract.

A more detailed analysis was carried out using high-performance liquid chromatography with diode array detection (HPLC-DAD) and assisted by chemometric analysis of the spectral data after chromatographic separation. Moreover, an acidic hydrolyzed form of the extract was also analyzed. Since the hydroethanolic extraction procedure is most suitable for phenolic compounds, such type of compounds were probed (mostly phenolic acids and flavonoids and their glycosides). The employed method allowed identification and determination of six compounds and they are listed in Table 1, together with the analytical performances of the method.

**Table 1:** Elution time, analytic parameters and found concentrations of the determined compounds in the studied samples.

No	Compounds	t_elution_ (min)	R^2^	LOD (μg/mL)	LOQ (μg/mL)	unhydrolysed extract (μg/mL)	SD (μg/mL)	hydrolysed extract (μg/mL)	SD (μg/mL)
1	chlorogenic ac.	6.32	0.997	4.3	13.0	1748	42	568	59
2	p-coumaric ac.	8.43	0.998	1.4	4.1	82	1	80	3
3	ferulic ac.	9.28	0.999	1.5	4.7	24	0.1	39	7
4	rutin	12.28	0.999	1.9	5.8	1604	10	91	0.1
5	isoquercitrin	12.94	0.999	2.1	6.3	308	8	168	10
6	quercetin	19.27	0.999	6.0	18.1	36	1	659	139

LOD–limit of detection, LOQ–limit of quantification, R^2^ –coefficient of determination for the calibration curves (at six levels of concentrations). SD represents standard deviation of the (n = 4).

**Fig 1:**
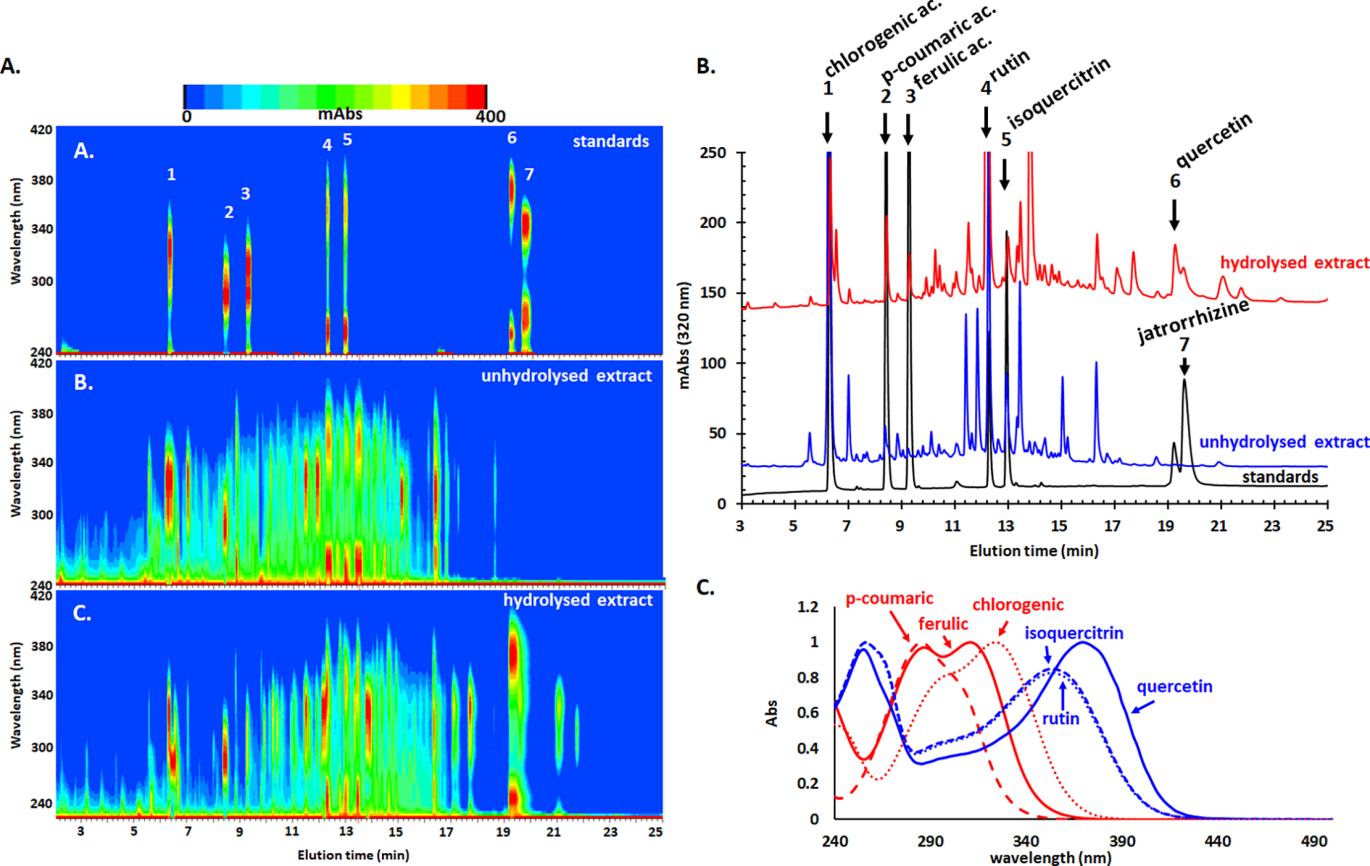
HPLC-UV-vis analysis of the unhydrolysed and hydrolysed *G verum* extract. (A) Heatmap of the chromatographic profile versus elution time. (**B**) Chromatograms of the two extracts and of the standards monitored at 320 nm. (**C**) UV-vis molecular absorption spectra of six of the employed standards.

The extract is highly rich in chlorogenic acid and rutin and contains important amounts of coumaric and ferulic acids and isoquercitrin and quercetin. The hydrolyzed extract contains much higher amount of quercetin originating from quercetin-based glycosides such as rutin, isoquercitrin and possible others. The chromatograms showed, however, a pool of many other compounds that potentially influence the quality and biological activity of the extract. A UV-vis heatmap of the chromatograms indicates indeed a change in the density from low retention time zone (more polar compounds, phenolic acids and glycosides) for the unhydrolysed extract towards high retention time zone (less polar compounds, hydrolysis generated aglycons) for the hydrolysed extract (Fig 1A). These heatmap patterns are also very useful for allowing to distinguish the spectral profile of the phenolics; phenolic acids present a single band (with some spectral shoulders) in the 250–380 nm region while flavonoids present two main bands, one in 320–420 nm region (band I) and one in 240–290 nm region (band II) (Fig 1A–1C). As expected, some of the compounds appear unchanged during hydrolysis.

**Fig 2:**
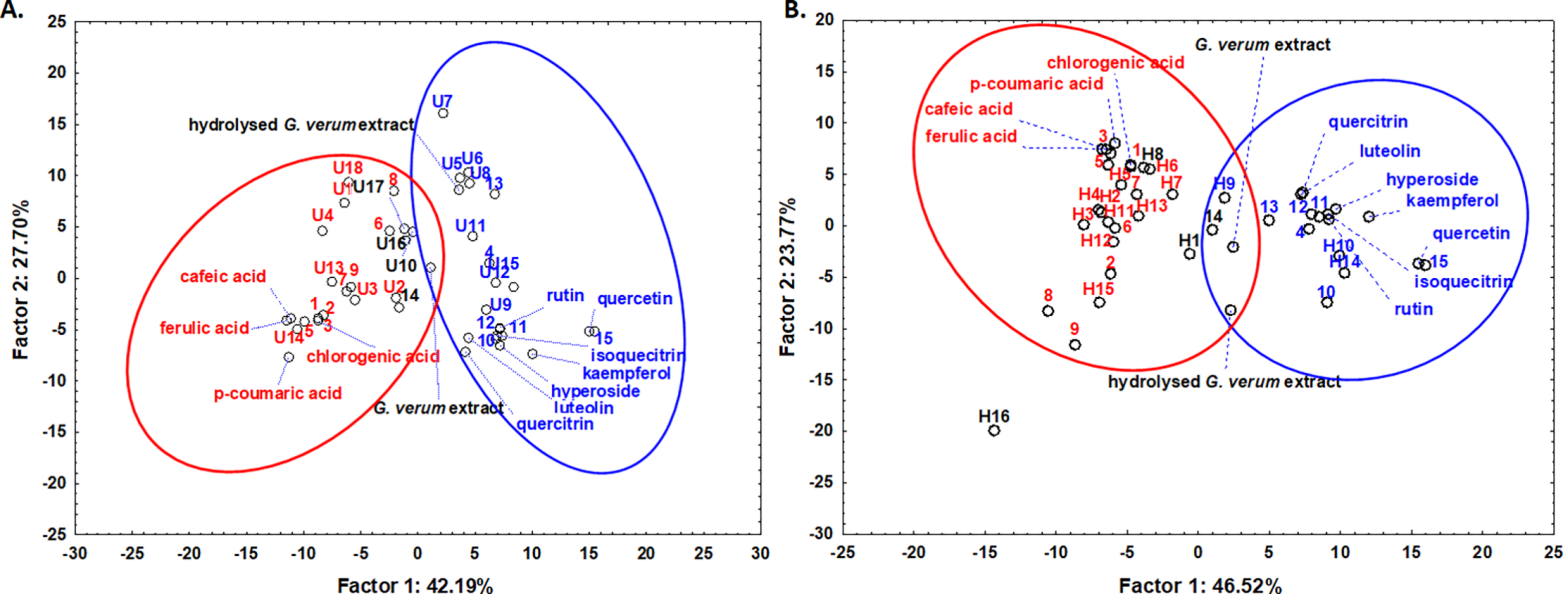
Phytoconstituent classification based on spectral similarities after chromatographic separation. Score plots of the first two principal components after applying PCA on the UV-vis mean spectra of the chromatographic peaks obtained by HPLC analysis of *G*. *verum* extract prior hydrolysis (**A**) and after hydrolysis (**B**). The UV-vis spectra of the two extracts, before separation and the standards are also indicated in both plots. The letter U stands for “unhydrolysed” and indicates compounds specific for this extract; the letter H stands for “hydrolysed” and indicates compounds found only in the hydrolysed extract, while the numbers lacking a letter indicate compounds that are found in both extracts. The number is attributed according to the retention order; the compounds are listed in Table 2.

The mean spectrum of each chromatographic peak that was satisfactorily separated from the near-by peaks and had a maximum of at least 10 mAbs was exported and chemometrically analyzed using PCA *after normalization* to exclude concentration effects. The analysis includes several standard spectra as controls. The PCA was applied on both sets of spectra, prior and after acidic hydrolysis; in both cases, two distinct groups could be identified in the scoreplots of the first two principal components (Fig 2). Based on the spectral features (maxima, position of bands, spectral shoulders), these two clusters proved to be phenolic acids on one side and flavonoids on the other side, all of the standards being correctly attributed. The spectra from the peaks belonging to the identified compounds were also positioned very close to the standard, indicating the high reliability of the analysis. Interestingly, the spectra of the extracts (both prior and after hydrolysis) were positioned in between the two clusters. The list of the compounds, their retention time and their classification in these two classes are listed in Table 2. The spectral similarity was also carefully checked by employing the built-in options of the Agilent software using the library containing a series of tens of phytochemicals. The compounds that had little similarity with any known member of either phenolic acid or flavonoid classes (less than 90%) were not attributed to any of the two classes and appeared colored in black in Fig 2.

**Table 2:** List of the compounds corresponding to each chromatographic peak (320 nm chromatogram) with their retention times (column 1) and their classification into two classes: Phenolic acids and flavonoids, based on spectral similarities*. Assuming comparable molar absorptivities at 320 nm for both classes, their relative content was roughly estimated using areas of the peaks at this wavelength.

Retention time (min)	Unhydrolysed extract		Hydrolysed extract		Observations, identification
	Label	%	Label	%	
5.54	U1	1.12	-	-	phenolic acid-like
5.58	-	-	H1	0.23	-
**6.27**	**1**	**32.60**	**1**	**3.6**	**chlorogenic acid**
6.54	-	-	H2	1.9	phenolic acid-like
6.63	U2	0.31	-	-	phenolic acid-like
7.01	2	2.56	2	0.37	phenolic acid-like
7.23	U3	0.48	-	-	phenolic acid-like
7.68	U4	0.55	-	-	phenolic acid-like
**8.40**	**3**	**2.02**	**3**	**2.03**	**coumaric acid**
8.89	4	1.20	4	0.38	flavonoid-like
**9.30**	**5**	**0.65**	**5**	**1.34**	**ferulic acid**
9.62	U5	0.65	-	-	flavonoid-like
10.19	6	0.65	6	0.35	phenolic acid-like
10.40	U6	1.31	-	-	flavonoid-like
10.42	-	-	H3	0.87	phenolic acid-like
10.53	-	-	H4	0.87	phenolic acid-like
10.60	U7	1.06	-	-	flavonoid-like
11.05	-	-	H5	1.17	phenolic acid-like
11.26	U8	1.61	-	-	flavonoid-like
11.46	7	4.31	7	2.15	phenolic acid-like
11.64	8	1.07	8	0.92	phenolic acid-like
11.88	9	4.50	9	0.86	phenolic acid-like
12.17	-	-	H6	19.4	phenolic acid-like
**12.53**	**10**	**12.14**	**10**	**0.97**	**rutin**
**12.81**	**11**	**3.93**	**11**	**0.87**	**isoquercitrin**
12.95	U9	1.27	-	-	flavonoid-like
13.34	-	-	H7	1.32	phenolic acid-like
13.45	12	5.07	12	2.9	flavonoid-like
13.80	U10	0.78	-	-	flavonoid-like
13.82	-	-	H8	11.7	flavonoid-like
14.00	U11	0.87	-	-	flavonoid-like
14.37	-	-	H9	1.36	flavonoid-like
14.39	U12	1.11	-	-	flavonoid-like
14.46	-	-	H10	1.07	flavonoid-like
14.77	13	0.23	13	0.58	flavonoid-like
14.76	-	-	H11	0.89	-
15.05	U13	2.23	-	-	phenolic acid-like
15.23	U14	0.75	-	-	phenolic acid-like
16.01	U15	0.10	-	-	flavonoid-like
16.23	U16	3.23	-	-	flavonoid-like
16.33	14	0.44	14	1.93	flavonoid-like
17.09	-	-	H12	1.63	phenolic acid-like
17.71	-	-	H13	1.91	phenolic acid-like
18.57	U17	0.51	-	-	flavonoid-like
**19.27**	**15**	**0.13**	**15**	**2.77**	**quercetin**
19.59	-	-	H14	1.96	-
20.92	U18	0.31	-	-	phenolic acid-like
21.07	-	-	H15	1.71	phenolic acid-like
21.76	-	-	H16	0.72	-

* Identified compounds are marked in bold. The letter U stands for “unhydrolysed” and indicates compounds specific for this extract; letter H stands for “hydrolysed” and indicates compounds found only in the hydrolysed extract, while the numbers lacking a letter indicates compounds that are found in both extracts. The number is attributed according to the retention order

### Antioxidant and pro-oxidant activities

Compared to other plant extracts analysed in our laboratory so far, *G*. *verum* appears to have a distinctly higher activity if evaluated with DPPH bleaching assay (4.6 mg quercetin equivalents/ g plant material) compared with TEAC method (0.082 mg quercetin equivalents/g plant material). This means that most of the chemical constituents act better in a more hydrophobic medium or associated with hydrophobic cell compartments such as membranes as previously discussed for such cases [22].

**Fig 3:**
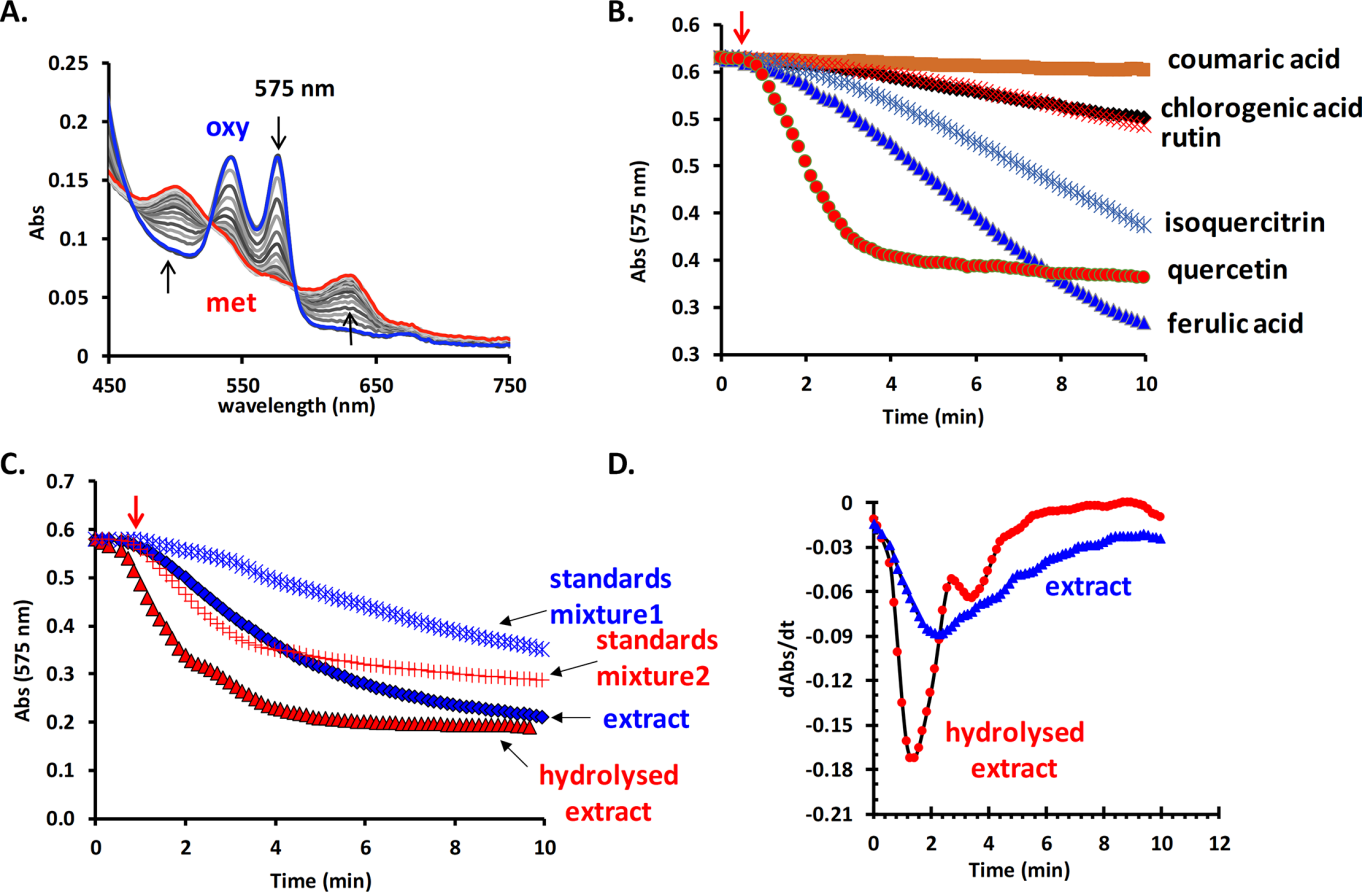
Evaluation of the pro-oxidant activity of the studied extract. The oxyHb (25 μM) is readily oxidized into met form in the presence of the extract (0.2 μg/mL final concentration) and laccase (100 nM) (**A**). Comparison between the prooxidant reactivity of the identified components of the extract all at the same molar concentration (5 μM) (**B**), their mixture in same ratios as in the extract prior hydrolysis (standard mixture 1) and after hydrolysis (standard mixture 2) and of the two analysed extracts (**C**). The comparison of the kinetic profile of the oxyHb oxidation in the presence of the two extracts and laccase depicted as first derivative of the measured curves (**D**).

Surprisingly, the pro-oxidant reactivity is much higher than the average of other studied extracts with the same method (Mot *et al*., 2014). The oxyHb (25 μM) is readily oxidized to the met form by only 0.2 μg/mL extract (Fig 3A). Among the identified components of the extract, quercetin appears to exhibit the highest prooxidant reactivity (Fig 3B). This is supported by the evidence that the hydrolysed extract, which has a much higher content of quercetin, is also more reactive (Fig 3C). It was previously shown that quercetin could easily generate radicals enzymatically therefore having significant pro-oxidant reactivity, at least in the hemoglobin assay (Mot *et al*., 2014). The first derivative of the kinetic profile of the oxyHb oxidation (Fig 3D) presents different local minima marking the different moment of the highest concentration of laccase-induced radical.

**Fig 4:**
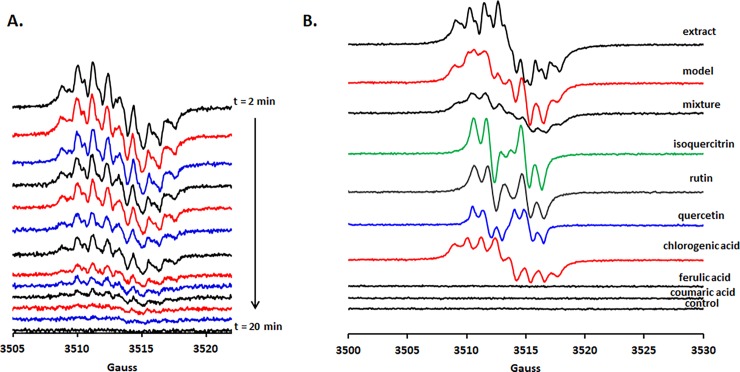
Probing the polyphenolic components and alkali-generated radicals reactivity in the studied extract using EPR spectroscopy. **A.** Time dependence of such generated radicals between 2 min (dead time due to spectrometer calibration prior measuring) and 20 min, at about 1.8 minutes interval. **B.** The dominant spectral fingerprint of the chlorogenic acid is visible in the extract with minor contributions of rutin and quercetin. Ferulic and coumaric acids gives no EPR spectrum while treated with sodium hydroxide as described in experimental section. The best fit for the EPR spectrum of the extract was obtained by a linear combination of the identified polyphenols in a 1/30/40 ratio (quercetin/rutin/chlorogenic acid) (model).

It was previously shown that a direct relationship exists between the total phenolic content of plant extracts, and the intensity of the signal detected using EPR for an alkaline-treated sample of the sample [14]. Moreover, the intensity and decay kinetics of these EPR signals can be significantly different between various polyphenolics. In the mentioned conditions (5 times diluted extract), the *G*. *verum* extract exhibits a well-resolved EPR spectrum indicating a high amount of polyphenols with distinct domination of chlorogenic acid, in good agreement with chromatographic results. On the other hand the fast radical decay compared to other extracts [16] indicates a high quenching reactivity of this extract (either in an antioxidant or pro-oxidant way or both) (Fig 4A and 4B). Additionally, no significant change of the spectral lineshape is detected during decay, indicating that there is a good cross-talk between all radical components (Fig 4A) during the entire process. Interestingly, a mixture of the identified components, in exactly the same ratio and conditions as in the extract, did not give rise to a similar EPR spectrum, indicating the importance of the other many minor components dramatically influence the reactivity (Fig 4B). Changing the reactivity of the extract by hydrolysis is of great importance since it was shown that phytoconstituents such as rutin are hydrolysed by the cecal microflora before absorption and thus changing its biological activity [23].

### Serum biochemistry

**Fig 5:**
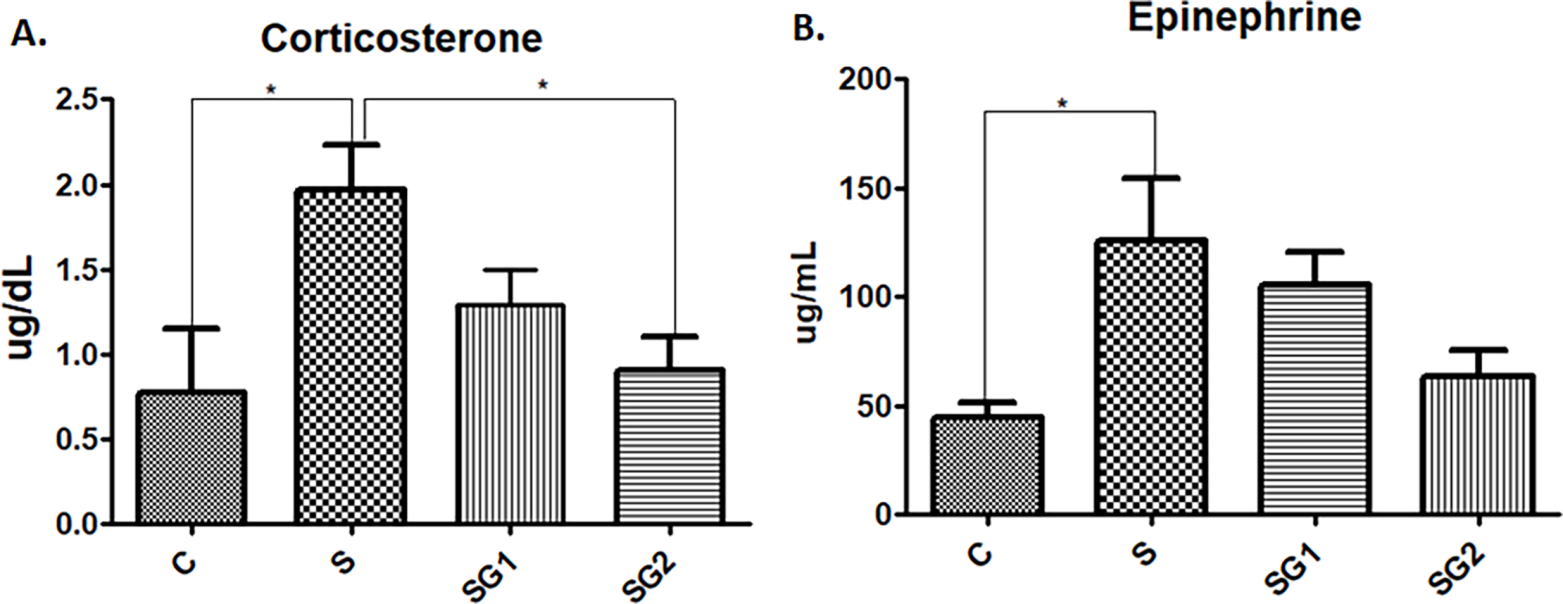
Hormone determinations in control and experimental animals. The effect of restraint and darkness stress on epinephrine and corticosterone levels (mean±SEM, n = 6), (**A**) C = 0.434 μg/dL; S = 1.96 μg/dL; SG1 = 1.29 μg/dL; SG2 = 0.90 μg/dL; (**B**) C = 44.4 μg/mL; S = 125.7 μg/mL; SG1 = 106 μg/mL; SG2 = 63.7 μg/mL; * Significant at *p* < 0.05 (t-test).

Exposure to acute restraint stress combined with darkness, resulted in elevated plasma corticosterone (RS; p<0.05); the *G*. *verum* groups (SG1 and SG2) clearly appeared to have a dose-dependent decrease in corticosterone levels. On the other hand, the plasma epinephrine levels upon exposure stress conditions had a corticosterone-like behaviour, i.e., a significant increase (p<0.05) as compared with control group. The extract adjusted the epinephrine levels in a dose-dependent manner, as represented in Fig 5. Furthermore, differences were found between the two distinct doses of extract (SG1 and SG2), as seen in Fig 5, in which the antioxidant-rich dose had remarkably and statistically-significant reduced the hormonal levels much more than the other one (RSG1).

The effects of repeated stress on the antioxidant activities of CAT, SOD, alongside with TBARS, markers of the oxidative stress, are depicted in Table 3. The activities of CAT and SOD were significantly (p<0.05) decreased in restraint stress as compared with Control group. Besides, the TBARS level was significantly (p<0.05) increased in stress alone as compared with control values. Treatment with *G*. *verum* extract determined a significant (p<0.05) increase of antioxidant enzyme activity as compared to the S group. Upon exposure to a higher dose of *G*. *verum* extract, the TBARS level significantly decreased as compared to the S group (p<0.05). In all the cases, there were dose-dependent variations.

**Table 3:** Oxidative stress parameters of control and experimental animals. Values are expressed as mean ± SEM.

Parameters	Control	S	SG1	SG2
CAT(U/mL)	56.36±4.10	41.51±5.99^*^	56.95±3.14^#^	61.01±1.21^#^
SOD (USOD/mL/min)	0.80±0.00	0.68±0.01^*^	0.82±0.01	0.85±0.00^#^
TBARS (nmol/mL)	4.43±0.47	8.06±1.26^*^	4.74±0.67	3.73±0.69^#^

* Significant at *p* < 0.05

** Significant at *p* < 0.01

*** Significant at *p* < 0.001 (compared with Control)

# Significant at *p* < 0.05

## Significant at *p* < 0.01

### Significant at *p* < 0.001 (compared with RS).

Similar patterns were noted in the circulating levels of biochemical markers of control and treated groups (Table 4). The levels of cholesterol (p<0.05) and creatinine (p<0.001) were significantly elevated during stress alone as compared with control values, while exposure to *G*. *verum* reduced the level of cholesterol; the strongest decrease was observed in the SG2 group (p<0.01) in comparison to the S group. Furthermore, creatinine concentrations were almost normalized after both doses of *G*. *verum*, as seen in the SG1 and SG2 groups. TP and ALT activity did not suffer any changes after stress exposure, but there is a tendency of increase in AST activity. No significant differences were found in TP concentration in RS group (p>0.05) as compared to Control and SG1 group, except SG2, where an increased TP concentration (p<0.01) is noted. The AST activity followed the same trends as cholesterol level. Thus, the lowest activity was registered in SG2 group, significantly different (p<0.05) from the G group. As compared to the Control, ALT activity was significantly decreased (p<0.01) in both groups treated with the extract (SG1 and SG2), but a statistical significance compared to the S group was observed only in SG1 (p<0.05).

**Table 4:** Serum biochemistry of control and experimental animals. Values are expressed as mean ± SEM.

Parameters	Control	S	SG1	SG2
TP (g/dL)	12.96±2.48	11.44±6.61	11.22±1.37	8.60±2.06^**^^,^^#^
Chol (mg/dL)	83.50±12.59	135.0±9.80^*^	118.0±14.39	74.29±3.79^##^
AST (U/L)	221.0±10.88	243.0±8.00	235.2±13.68	166.7±10.93^#^
ALT (U/L)	86.0±0.00	90.0±1.95	54.0±5.37^**^^,^^#^	59.8±3.73^**^
Crea (mg/dL)	0.80±0.00	1.17±0.02^***^	1.20±0.05^***^	1.26±0.04^***^

* Significant at *p* < 0.05

** Significant at *p* < 0.01

*** Significant at *p* < 0.001 (compared with Control)

# Significant at *p* < 0.05

## Significant at *p* < 0.01

### Significant at *p* < 0.001 (compared with RS).

Exposure to stress situations can activate the hypothalamic-pituitary-adrenocortical axis, which plays a pivotal role in the stress response [24], leading to central and peripheral biochemical changes regarding oxidative stress markers, protein and lipid metabolism, liver and kidney and enzyme activities [25,26]. It is well known that oxidative stress is mainly caused by free radicals, which in turn can further favor several pathologies [27]. The antioxidant activity of *G*. *verum* extract has been studied previously using *in vitro* techniques such as the neutralization of DPPH radical, hydroxyl radicals (OH), hydrogen peroxide (H_2_O_2_) and also the inhibition of lipid peroxidation [1]. For the methanolic extract of *G*. *verum*, the free radical scavenging capacity (RSC) and protective effect against lipid peroxidation were previously reported [1]. Administration of dietary antioxidants to patients may help strengthen the cellular defense, removing ROS and prevent oxidations [28]. The intake of polyphenols in oxidative stress conditions may protect the organism by scavenging the harmful radicals or by protecting the cells strengthening their antioxidant barriers [29,30], [31,32]. Because of the phenolic nucleus and extended side chains, polyphenols easily form stabilized phenoxyl radical which may also contribute to their free radical scavenging effect [33].

An acute restraint stress model was used in our experiments combined with darkness, in order to disturb the cellular homeostatic balance and so to investigate and validate the protective and adaptogenic effects of *G*. *verum* extract. In addition, the restraint stress response could be slightly amplified by light/dark stress, as it is known form the literature that during dark, corticosterone is slightly increased as compared in the daylight [18]. Our study evaluates the antioxidant status of the *G*. *verum* extract and further provides insight into the biochemical alterations during acute restraint stress and dark conditions. Previous studies revealed that ferulic acid, chlorogenic acid, kaempferol, rutin are ubiquitous plant constituents that have positive effects in prevention of tissue damage by radical scavenging mechanisms [28,29,34]. Upon exposure to restraint stress, corticosterone and epinephrine levels were highly increased, as proof of an overactive adrenal gland; the antioxidant-rich extract was apparently strong enough to suppress the oxidative damage and so the hormone levels returned at a near normal value. It has been previously found that catecholamine levels and corticosterone are highly increased during swimming stress, but additional supplements based on chlorogenic acid had stress-reducing actions regarding blood corticosterone and catecholamines induced by stress [35]. There are studies that have shown an activation of the neuroendocrine components under stressful conditions that stimulate the sympathetic nervous system which leads to increased secretion of epinephrine, norepinephrine and corticosteone, which are the main stress markers associated with restraint conditions [24,36]. Besides, restraint stress also activates the autonomic nervous system which in association with liver and kidney damages lead to overproduction of ROS that may affect the biochemical parameters related to hepatic and kidney metabolism [25]. Thus, in accordance with our study, oxidative stress markers, such as serum CAT, SOD activities and TBARS level, as well as ALT and AST activities, total proteins concentration, cholesterol and creatinine concentrations, are significantly changed in restraint stress, as also previously reported [25,37–39].

Many reports have indicated that restraint stress induced an increase in oxidative stress markers [25,37,40]. Indeed, in the present study, 3 hours/day of restraint stress combined with light/dark stress caused significant oxidative damage as noticed by increased lipid peroxidation, which led to high concentration of TBARS, as well as in a reduced CAT and SOD activities. Oxidative stress markers such as MDA is highly increased in rats exposed to acute restraint stress and such changes does not appear only in plasma but also in tissues, which led to a series of negative signaling reactions[41]. Both of the *G*. *verum* extract doses used in our experiments improved the investigated clinical outcome, in particular the second dose (SG2). Our analysis showed that the extract contains antioxidant polyphenols and the foremost chlorogenic acid and rutin, likely responsible for the improved outcome of the clinical parameters. There are many studies that revealed well-marked outcomes regarding the antioxidant defense of chlorogenic acid by increasing the activities of SOD, CAT and suppressing lipid periodation *via* MDA, in liver and kidney of mice [42,43]. Moreover, upon undergoing oxidative stress the liver tissue has been exposed to lipid peroxidation which led to an increase of ALT and AST activity in serum, as well as cholesterol concentration. After exposure to stress, a high activity of hepatic transaminases represents a marker of liver damage in line with previous observations [38]. ALT and AST remain the most important indicators for the hepatic cells evaluation, which in our experiment, *G*. *verum* extract turned out to be highly effective by reducing the levels of these enzymes even lower than the control values. Additionally, decreasing of the hepatic enzymes appear infrequently and are not well understood [44], but lowering these enzymes after only 7 days of treatment with *G*. *verum* extract, definitely shows a quick regeneration of the hepatocytes. Also, SG2 has significantly decreased the serum cholesterol level, an effect which can be linked to the hypocholesterolemic effects of the chlorogenic acid. It was reported that chlorogenic acid is responsible for decreasing oxidation of low-density lipoproteins (LDL) and total cholesterol concentration [29]. Also, there are studies showing the efficiency of chlorogenic acid in stimulating hepatic fatty acid β-oxidation and suppressing the activities of HMG-CoA reductase in the liver [29].

Furthermore, an increased serum creatinine level is commonly found when kidney or skeletal muscle tissues are damaged [25]. All these effects could be the result of lipid peroxidation and a high amount of ROS. *Nota bene*, in the small intestine, chlorogenic acid is hydrolyzed to caffeic acid, which plays a more important role in renoprotection and against the I/R injury [32,45]. Hence, the amplified reactivity of the kidney due to the restraint background was subsequently markedly attenuated by the activity of supplementary antioxidants, mainly chlorogenic acid and rutin derivatives. Chlorogenic acid has been proven to be a remarkable antioxidant, that possesses a series of properties starting with antioxidant, anti-inflamatory and antiproliferative, that have been proven both *in vivo* and *in vitro* [32,46]. On the other hand, supplementation of the diet with natural antioxidants, such as chlorogenic acid and rutin derivatives, could be associated with therapeutic benefits against oxidative stress [47]. Both chlorogenic acid and rutin were recently shown to be important modulators in several stress conditions [48,49]. Additional investigations are needed to unravel the fine mechanisms of supplementary antioxidants. Hence, the amplified reactivity of the kidney on the restraint background could be caused by something else. A possible explanation could be supported by the pro-oxidant activity of quercetin, which has been proven to be a remarkable antioxidant, that possesses a series of properties starting with antioxidant and anti-inflamatory that have been proven both *in vivo* and *in vitro* [50]. Besides, supplementation of the diet with quercetin in chronic glomerula disease could be associated with adverse renal effects [47]. Moreover, early studies have given evidence that 1% of quercetin in the diet aggravated renal tubular necrosis in the LEC strain of rats prone to accumulate copper [47]. Therefore, our situation could be explained by the presence of quercetin on an oxidative stress background, but this idea needs to be further studied.

## Conclusions

To conclude, the present study indicates that *G*. *verum* extract is a valuable antioxidant which exhibits activity against the acute restraint/dark stress induced oxidative damages, mediated by its modulatory effect on the HPA axis. The biochemical profile was markedly improved after the treatment with the extract in acute restraint conditions. Overall, our study indicates an effective dose-depending medicine prototype, potentially able to restore the biochemical alterations during acute restraint stress conditions.

## Supporting information

S1 Fig HPTLC images of the silica gel plates, 60F_254_ developed with toluene: Acetone: Formic acid, 9:9:2 (v/v/v) as mobile phase. Derivatized with NP/PEG (**A.**) visible (**B.**) UV 254 nm (**C.**) UV 366 nm. Bands: 1 –ferulic acid, 2 –galic acid, 3 –chlorogenic acid, 4—quercetin, 5—rutin, 6—kaempferol, 7 –*G*.*verum* extract.
(DOCX)

## Abbreviations

ROS: reactive oxygen species
HPA: hypothalamic-pituitary-adrenal axis
CS: corticosterone
EP: epinephrine
RS: restraint stress
DPPH: 2,2-diphenyl-1-picrylhydrazyl
FC: Folin—Ciocâlteu
PEG: polyethylene glycol
SOD: superoxide dismutase
CAT: catalase
GPX: glutathione peroxidase
HPTLC: high performance thin layer chromatography
ALT: alaninaminotranferase
AST: aspartat aminotransferase
TE: trolox equivalent
TP: total proteins
Crea: creatinine
TBARS: thiobarbituric acid reactive substances
Chol: cholesterol
