# Supplementary material for: Chemo-mapping and biochemical-modulatory and antioxidant/prooxidant effect of Galium verum extract during acute restraint and dark stress in female rats
Source: PLoS One. 2018 Jul 3;13(7):e0200022. doi: 10.1371/journal.pone.0200022 (PMC6029781; doi:10.1371/journal.pone.0200022)
Supplement: S1 Fig — Derivatized with NP/PEG (A.) visible (B.) UV 254 nm (C.) UV 366 nm. Bands: 1 –ferulic acid, 2 –galic acid, 3 –chlorogenic acid, 4—quercetin, 5—rutin, 6—kaempferol, 7 –G.verum extract. (DOCX) [file pone.0200022.s001.docx]

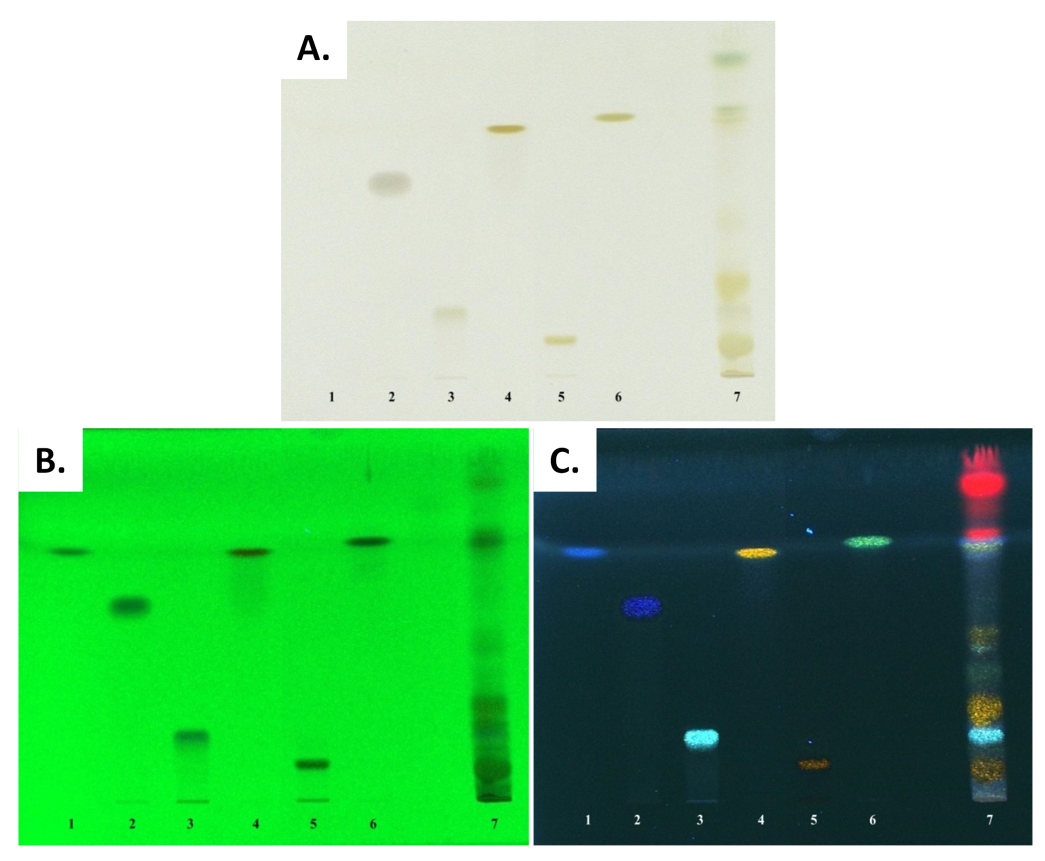


**S1 Figure.** HPTLC images of the silica gel plates, 60F_254_ developed with toluene: acetone: formic acid, 9:9:2 (v/v/v) as mobile phase. Derivatized with NP/PEG (**A.**) visible (**B.**) UV 254 nm (**C.**) UV 366 nm. **Bands:** 1 –ferulic acid, 2 – galic acid, 3 – chlorogenic acid, 4 - quercetin, 5 - rutin, 6 - kaempferol, 7 – *G.verum* extract.
